# Investigating Forkhead Box O Transcription Factor 1 Gene’s Relation to Immunoglobulin E in House Dust Mite-Allergic Asthma Patients

**DOI:** 10.3390/arm91060039

**Published:** 2023-11-15

**Authors:** Rania A. Mohamed, Ahmed ElSadek Fakhr, Shereen A. Baioumy

**Affiliations:** 1 Department of Biology, Deanship of Educational Services, Qassim University, P.O. Box 5888, Unaizah 56219, Qassim, Saudi Arabia; 2 Department of Parasitology, Faculty of Veterinary Medicine, Zagazig University, P.O. Box 44519, Zagazig 44516, Egypt; 3 Medical Microbiology and Immunology Department, Faculty of Medicine, Zagazig University, P.O. Box 44516, Zagazig 44519, Egypt; afakhr@imc.med.sa (A.E.F.); sabaioumy@medicine.zu.edu.eg (S.A.B.); 4 Laboratory Pathology and Blood Bank, International Medical Center, P.O. Box 21589, Jeddah 23214, Makkah, Saudi Arabia

**Keywords:** HDM, allergic asthma, gene expression, FoxO1, SIRT1, total IgE

## Abstract

**Highlights:**

**What are the main findings?**

- Subcutaneous immunotherapy is a long-term effective immunotherapy;
- Immunity biomarkers such as IgE, FoxO1, and Sirtuin 1 are important biomarkers that have potential roles in the pathogenesis of asthma and remission of clinical symptoms.

**What is the implication of the main finding?**

- There is correlation between IgE, FoxO1, and Sirtuin 1;
- Further broader-scale studies are needed to determine a novel protocol for the control and remission of clinical symptoms of HDM-allergic asthma.

**Abstract:**

House dust mite (HDM)-allergic asthma is an abnormal immune response to extrinsic aeroallergens found in human vicinities. Studying the role of the associated immunity biomarkers and their interplay helps in discovering novel therapeutic strategies that can be used in adjunct with effective long-term immunotherapy. This study investigates the total serum IgE, FoxO1, and Sirtuin 1 (SIRT1) gene expressions in HDM-allergic asthma patients. We enrolled 40 patients for each of the following three groups: an HV group of healthy volunteers and HDM/AA and HDM/SCIT groups of HDM-allergic asthma patients who did not and who did receive immunotherapy before recruitment in this study, respectively. The results elucidated that total IgE was strikingly elevated in the HDM/AA group and showed little decline in the HDM/SCIT group. Both FoxO1 and SIRT1 gene expressions showed the highest levels in the HDM/SCIT group. There was a negative correlation between total IgE and both FoxO1 and SIRT1 in the HDM/AA group while there was a positive correlation with SIRT1 in the HDM/SCIT group. In conclusion, the interplay of the three immunity biomarkers related to HDM-allergic asthma after the course of immunotherapy treatment suggests further, broader studies on the feasibility of their role as immunity biomarkers in the control and remission of HDM-allergic asthma.

## 1. Introduction

House dust mites (HDM) are a prominent reason for respiratory allergies, as HDM allergens are the most widespread respiratory sensitization chemicals linked to allergic asthma and rhinitis [1] House dust mites are a kind of nonpathogenic arachnid parasite that are found in human and animal habitations as well as in medicinal herbs and stored products. Their populations are concentrated in coastal areas, the tropics, and subtropics due to higher temperatures and humidity. The mites’ feces are the main source of the mites’ aeroallergens. HDM-allergic asthma is a chronic worldwide disease that is manifested by airway inflammation with varying degrees of symptoms. It has a great influence on the quality of human life. The disease is due to the body’s immune reaction to several types of aeroallergens. House dust mite-allergic asthma is one of the most common types of asthma among the world’s population and affects from about 25% to 30% of humans all over the world [2]. Avoiding environmental exposure to these allergens is usually unsuccessful due to the great aerodynamic properties of HDM [3]. In addition to environmental factors, the dose, type of allergen, route, consistency of exposure and genetic predisposition and airway responsiveness of the human subject are important reasons for individual variations in immune response. Although there are different types of pharmaco- and immunotherapies used to control HDM-allergic asthma symptoms, only immunotherapy can provide long-term relief through modulation of the immune reaction.

Immunoglobulin E (IgE) is one of the five classes of antibodies that are responsible for specific humoral immunity. IgE is involved in parasitic worm infestations and allergic reactions such as asthma and hay fever. It is the cardinal sign of type 1 hypersensitivity [4]. The importance of IgE in the pathobiology of asthma was not under great consideration until the release of anti-IgE therapy [5]. Whereas the values of total IgE greatly overlap between allergic and nonallergic subjects, its serum level is closely correlated to the risk of asthma [2]. The findings of Burrows et al. challenged the earlier concept of the existence of basic differences between allergic and nonallergic types of asthma [2]. Independently of continuous allergen exposure, IgE responses underlie disease persistence that explains the remission of only 30% of patients after absolute avoidance of the causative allergen in occupational asthma [6].

Forkhead box O (FoxO) transcription factor 1 is a SIRT1 target. FoxO1 affects many white blood cells, such as neutrophils and macrophages, that have powerful phagocytic functions, in addition to the regulatory T cells and B lymphocytes that are responsible for eliciting specific immune responses, as well as skin cells such as keratinocytes and mucosal dermis [7]. These cells have crucial roles in the response to oxidative stress, DNA repair, cellular metabolism, and homeostasis [8]. The location of FoxOs determines if they are functional or not. The activation of their target genes necessitates their expression inside the nucleus. However, if they are translocated to the cytoplasm by the growth factor, they are not functional [9]. The deacetylation activities of FoxOs are regulated by Sirtuin 1 (SIRT1).

One of the most important members of the family of silent information regulators (Sirtuins) is Sirtuin 1 (SIRT1). It is a nicotinamide adenosine dinucleotide (NAD), a dependent protein that deacetylases different histones and non-histones. It has a multitasking role in regulating the immune system and maintaining homeostasis [10]. SIRT1 has an important modulating role in a variety of pathological and physiological processes. It inhibits cellular apoptosis, inflammation, oxidative stress, and neurodegeneration and regulates metabolism and autophagy [11,12]. Moreover, it acts as a critical enzyme that can increase the life span of some organisms such as yeast, some metazoans, and mice [13].

SIRT1 and FoxO1 interactions and their effects on different diseases have been studied in many research papers that define them as potential targets of immunotherapy. Some of these research papers studied the relationship between SIRT1 expression and asthma conditions. SIRT1 activation exerts an anti-inflammatory effect due to the inhibition of IL-6 and IL-8 and leads to improvements in the asthma inflammatory response [14]. On the other hand, its activation may exert a pro-inflammatory role due to the inhibition of certain substrates [10]. Overexpression of SIRT1 and suppression of FoxO1 cause tumor growth and increase cell survival of cancer cells [15,16]. The controversial role of SIRT1 in regulating asthma and other respiratory diseases can be attributed to the abundance of substrates and their great variability, in addition to the involvement of different regulatory pathways [10]. The roles of SIRT1 and FoxO1 and their interactions in different types of diseases such as tumor progression, toxoplasmosis, and asthma are not completely elucidated [17]. In this work, we aim to find out more about the following three immunity biomarkers: total serum IgE, gene expression of SIRT1, and FoxO1, and investigate their relations to total serum IgE during house dust mite-allergic asthma disorder. This investigation allows the use of the potential biomarkers in early intervention to prevent both morbidity and mortality due to allergic diseases.

## 2. Patients and Method

### 2.1. Study Design and Ethical Considerations

Eighty HDM-allergic asthma patients, who visited the allergy and immunity unit, faculty of medicine, Zagazig University, Egypt, were allocated to one of two study groups depending on whether they were treated with subcutaneous immunotherapy or were not treated with any kind of treatment at all. In addition, forty healthy volunteers were recruited in the control group of this study from December 2021 to May 2022.

This study was approved by the Zagazig University Institutional Review Board (IRB), Egypt. The approval number is 8097-3-10-2021. This study was conducted in accordance with the Declaration of Helsinki and written informed indications of consent were signed and collected from each subject.

#### 2.1.1. Sample Size

The sample size was calculated using the Open-Epi program with a confidence level of 95% and power of 80% as the mean total IgE in the asthmatic group was 222.3+/−203.4 compared to 84.4+/−18.6 in the control group [18]. The calculated sample size was 36 but we increased the total number of participants to 120 to increase the power (chance of detection) to 80% and the confidence level to approximately 95%, which allowed our results to reach the determined level of significance. The participants were allocated equally into 3 groups: the healthy volunteers’ group (HV), the HDM-allergic asthma (HDM/AA) group for those patients who did not receive any treatment, and the HDM/SCIT group for those patients who received subcutaneous immunotherapy for the past six months before enrollment in this study.

#### 2.1.2. Inclusion and Exclusion Criteria

The subjects who were included in this study were from both sexes. The subjects of the allergy groups were positive for the skin prick test and specific HDM IgE. They were tested for pulmonary function (obstructive pattern) and low FEV1/FVC.

Subjects who were excluded from the study were those who had any other type of allergy, autoimmune disease, immunodeficiency disorder, or any other respiratory or cardiovascular disease including chronic obstructive pulmonary disease (COPD), as well as the cancer patients, pregnant women, smokers, and those who declined to participate.

In addition to the inclusion criteria, each subject provided a full detailed history including demographic data, prior clinical examinations of the respiratory system, and chest X-rays to exclude any other pulmonary pathology.

#### 2.1.3. Assessment of Severity of Asthma

Asthma severity was graded from the mildest to the most severe (I–IV). Grading of asthma was based on a follow-up with patients six months post-enrollment, which addressed symptoms throughout the day, the effect of asthma on the quality of life, the use of corticosteroids, and expiratory flow rate according to GINA guidelines 2020 [19].

#### 2.1.4. Pulmonary Function Test

Spirometry was measured using a computerized spirometer (Jaeger MasterScreen™ IOS, version 5.2 manufactured by VIASYS Healthcare GmbH, Hoechberg, Germany). The measured forced expiratory volume in 1 s (FEV1), forced vital capacity (FVC), and their ratio expressed as a percentage were measured. Ratios higher than 80% were considered normal.

#### 2.1.5. Skin Prick Test

According to the methodology of Bernstein et al., a skin prick test was performed using standardized allergen extracts for positive HDM-allergic asthma patients. Histamine dihydrochloride (10 mg/mL) was used as positive control and saline as *negative* control [20]. Allergen extracts for aeroallergens (house dust mites: *Dermatophagoides pteronyssinus, D. farinae*, grass, mixed pollens, mixed molds, tobacco, cotton, wool, cockroach, and hay dust) were provided by Hamilton (Omega, Allergy OVERSEAS consultant Inc., Hamilton, ON, Canada). Wheels of ≥3 mm diameter were considered positive [21].

### 2.2. Samples

Under complete aseptic conditions, serum and whole blood samples were collected from all subjects at enrollment. Serum samples were stored at −20 °C for further total IgE measurement. Whole anticoagulated blood samples were sent immediately for further FoxO1 and Sirtuin 1 gene expression analysis.

### 2.3. Measurement of the mRNA Expression Levels of FoxO1 and Sirtuin 1

#### 2.3.1. Separation of Peripheral Blood Mononuclear Cells (PBMCs)

Peripheral blood mononuclear cells (PBMCs) were separated using Ficoll-Hypaque (Ficoll solution, Sigma, MA, USA) density gradient centrifugation from the EDETA anticoagulated blood samples within 2 h of sample collection.

#### 2.3.2. RNA Extraction and Isolation

The PBMCs were isolated by gentile aspiration and immediately subjected to RNA extraction using the RNA Purification Kit of Thermo Scientific GeneJET according to the manufacturer’s instructions. Total RNA was instantly preserved at −80 °C until it was used.

Total RNA concentration was measured by using the Quantus™ RNA system on a Quantus fluorometer (Promega, Madison, WI, USA). Each sample was homogeneously diluted by adding sterile distilled water according to total RNA concentration.

#### 2.3.3. Synthesis of the First-Strand Complementary DNA

Thermo Scientific’s reverse-transcription RevertAid First Strand cDNA Synthesis Kit was used to synthesize the first-strand complementary DNA following the manufacturer’s instructions.

#### 2.3.4. Quantitative Analysis of FoxO1, SIRT1, and 18S rRNA Genes

Thermo Scientific’s Maxima SYBR Green qPCR Master Mix on the Step One Real-Time PCR System (Applied Biosystems Technologies, Waltham, MA, USA) was used to perform a quantitative RT-PCR assay to determine the mRNA expression levels of FoxO1, SIRT1, and 18S rRNA, which was used as a reference gene.

The primer sequences and thermocycler conditions are listed in Table 1 and Table 2, respectively, followed by a melting curve cycle.

The comparative threshold cycle (ΔΔCt) method was used for relative quantification of the target mRNA. Each reaction was conducted in duplicate. The expression level of the target mRNA was normalized by the 18S rRNA expression level [8]. RNA samples that were not treated with reverse transcriptase were used as negative control to ensure the absence of DNA contamination.

### 2.4. Enzyme-Linked Immunosorbent Assay

The quantitative measurement of total serum IgE levels was performed using a commercially available enzyme-linked immunosorbent assay (ELISA) kit supplied by Thermo Fisher Scientific, Inc. (Invitrogen IgE Human ELISA Kit Catalog Numbers BMS2097, Carlsbad, CA, USA), according to the manufacturer’s instructions. The assay range was 7.8–500 ng/mL. The IgE concentrations in the healthy, nonatopic test subjects were greatly dependent on age. The recommended threshold value for adults is 240 ng/mL [25]. The absorbance of standards and samples was measured at 450 nm using a microtiter plate ELISA reader (Biotek, Winooski, VT, USA).

### 2.5. Statistical Analysis

GraphPad Prism 9.0 software (http://www.graphpad.com/scientific-software/prism/ ^®^ Statistics 26.0 accessed on 17 June 2023) for Windows was used for statistical analysis and graphs. A normality test was performed initially using Kolmogorov–Smirnov and Shapiro–Wilk tests. One-way ANOVA and Kruskal–Wallis tests were used to compare groups. The correlations between FoxO1, SIRT1, and total IgE were studied using Pearson correlation. Results are expressed as mean ± standard deviation and as numbers or percentages. A *p* value ≤ 0.05 is considered statistically highly significant.

## 3. Results

### Participants Criteria

The clinical features and study participants’ criteria are listed in Table 3. Each group had 40 participants. The age range for all groups was higher than 16 and lower than 58 years old. There was no significant difference in the mean age. Roughly half of the participants were female (50% in the HDM/SCIT group, slightly more in the other two groups). The majority of the case study participants lived in urban areas. It was clear that most HDM-allergic asthma patients had a family history of asthma (80% and 77.5% in HDM/AA and HDM/SCIT groups, respectively), while most of the healthy volunteers had no family history of asthma.

**Figure 1:**
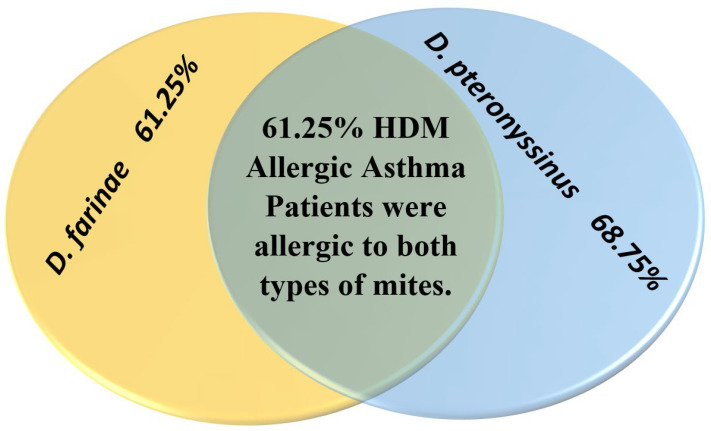
The percentage of HDM allergies in all HDM-allergic asthma patients.

As an indicator of the prevalence of HDM species, when we combined both HDM/AA and HDM/SCIT groups regarding the sensitivity to either one of the mite species or both of them, it was found that most (68.75%) allergic asthma patients (28 + 27= 55 out of 80 HDM-allergic patients) were allergic to *D. pteronyssinus*, while 61.25% (24 + 25 = 49 out of 80 HDM-allergic patients) were allergic to *D. farinae* and many of them (61.25%) were allergic to both species (Figure 1).

**Figure 2:**
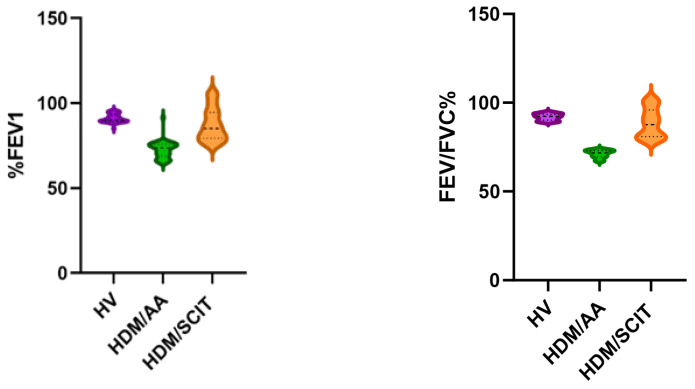
Pulmonary function test results of the study groups.

Regarding asthma severity and pulmonary function test, the allergic patients who did not receive SCIT had varying degrees of asthma ranging from very mild to moderate asthma. The associated mean FEV1/FVC% was 71.14 ± 2.141. However, the allergic patients who received SCIT had milder asthma and the associated mean FEV1/FVC% was 88.59 ± 8.247, which was closer to the mean FEV1/FVC% of healthy volunteers (91.91 ± 1.849). The differences in the pulmonary function test results between the three groups were highly significant. However, there was no significant difference between the HV and HDM/SCIT groups, as shown in Table 4 and Figure 2.

**Figure 3:**
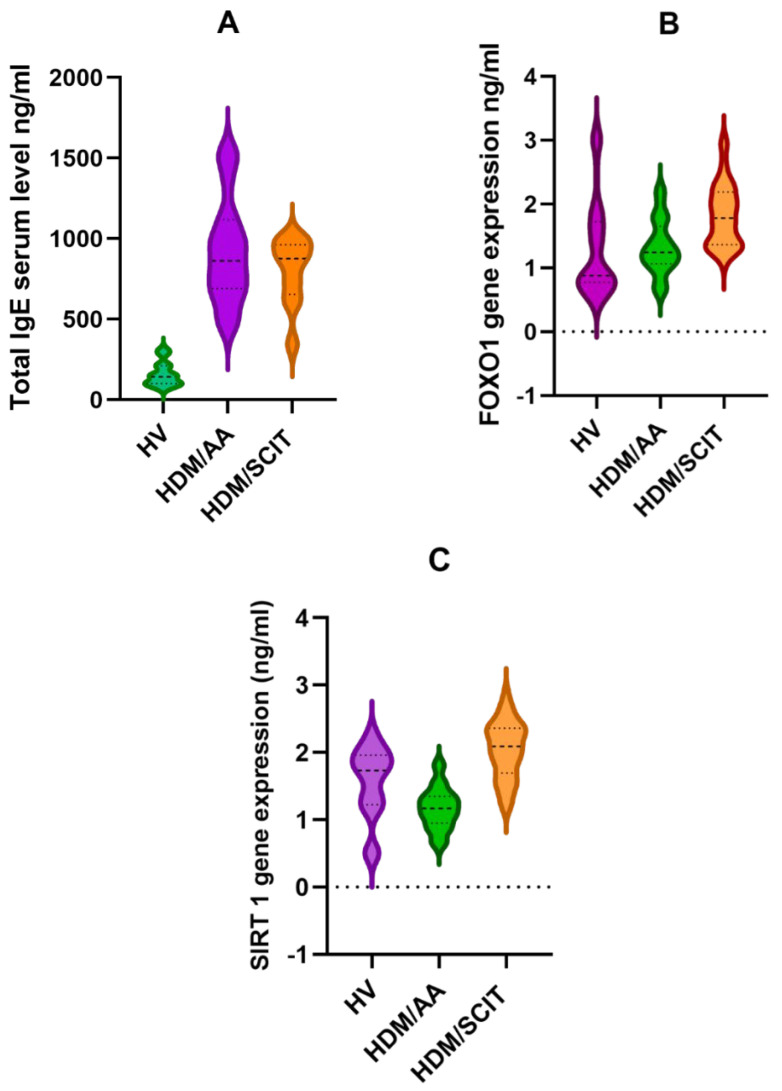
Comparison of total IgE levels and FoxO1 and SIRT1 gene expressions between the study groups. A: Total serum IgE level of the study groups, B: FoxO1 gene expression of the study groups C: Sirtuin 1 gene expression of the study groups.

Concerning the total serum IgE levels, they were highly elevated (921.3 ± 317.3) in the HDM/AA group and declined to 798.2 ± 204.0 in the HDM/SCIT group, which was still very far from that of the HV group (158.3 ± 73.11). However, there was no significant difference between HDM/AA and HDM/SCIT groups in total serum IgE levels; the statistical difference was highly significant between the HV group and the other two groups, as shown in Table 4 and Figure 3A.

Upon comparing the FoxO1 gene expression level, HDM-allergic asthma had an impact on FoxO1 gene expression that increased significantly from 1.29 ± 0.756 for the HV group to 1.822 ± 0.485 for the HDM/SCIT group. Although its level increased slightly in the HDM/AA group (1.326 ± 0.426), there was no significant difference when compared to the HV group (Table 4 and Figure 3B).

SIRT1 gene expression was affected by the allergic condition and declined significantly from 1.569 ± 0.5210 in the HV group to 1.168 ± 0.3074 in the HDM/AA group. As a positive impact of SCIT, the level of SIRT1 highly increased to 2.039 ± 0.4279 in the HDM/SCIT group. The difference between the three groups was highly significant as shown in Table 4 and Figure 3C.

**Figure 4:**
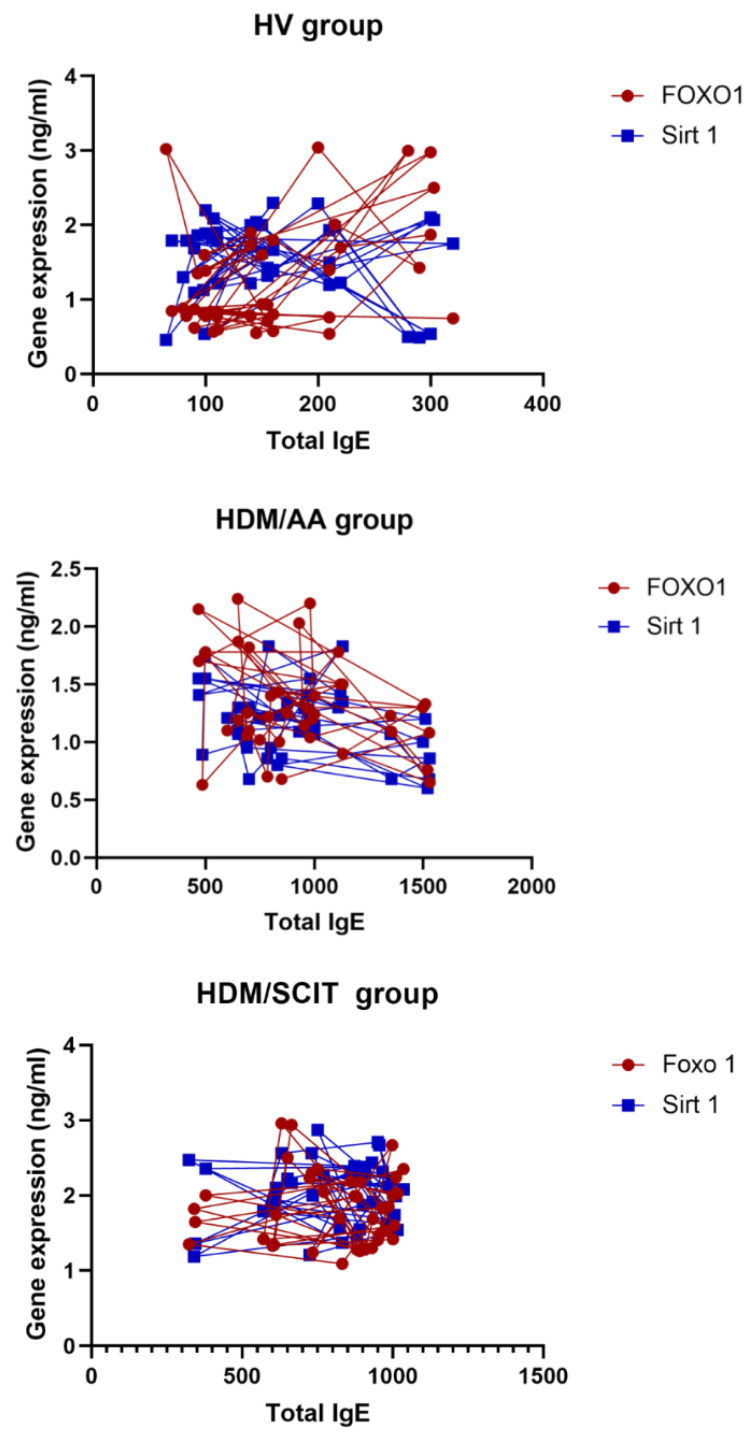
Spearman correlation analysis between total serum IgE levels and FoxO1 and SIRT1 gene expression in different study groups.

By studying the Spearman correlation of total serum IgE levels and FoxO1 and SIRT1 gene expression, it is concluded that total serum IgE levels were positively correlated with FoxO1 in the HV group while they were negatively correlated in the other HDM/AA and HDM/SCIT groups (Table 5 and Figure 4). On the other hand, total serum IgE levels were negatively correlated with SIRT1 in HV and HDM/AA groups while they were positively correlated in the HDM/SCIT group.

## 4. Discussion

A biomarker was previously defined as an objectively measurable criterion that can be evaluated and used as an indicator of physiological or pathological processes or pharmacological response to a therapeutic intervention. A common example is cholesterol which is associated with the development of cardiovascular diseases. Increasing the knowledge about potential biomarkers is valuable in allowing early intervention to prevent both morbidity and mortality arising from allergic diseases. Due to the limitation of data, more investigations are needed to identify these biomarkers and their combinations [13,26,27]. The significance of this study is based on the investigation of different immunity biomarkers, their interplay, and their relations in HDM-allergic asthma disorder. These data can add to the knowledge and affect future planning for asthma therapy. However, house dust mites are free-living noninfectious arachnids. The digestive enzymes secreted in their feces have a great impact on human health. HDM-allergic asthma is one of the most common allergic and chronic diseases globally. It is caused by exposure to dust mites’ aeroallergens which are extensively found in homes and house dust. Most homes are either coinhabited with both *Dermatophagoides pteronyssinus and D. farinae* (81.7%) or inhabited with a single species (75%) [28]. *D. pteronyssinus* (98%) is more dominant than *D. farinae* (83%) [29]. In this study, we obtained similar results, as more HDM-allergic patients were allergic to *D. pteronyssinus* (68.75%) compared to those allergic to (61.25%) *D. farinae*. In addition, most of the allergic asthma patients were allergic to both types of mites (61.25%). Most allergic asthma patients, whether they were treated with SCIT (77.5%) or not (80%), had a family history of asthma that can be attributed to the genetic predisposition to asthma. Previously published research papers have studied the predisposing factors to asthma and explained that it is expected that patients with a family history of asthma suffer from asthma as it is a polygenic disorder that is controlled by many factors including interacting genes and environmental factors. The genetic factor contributes to either protection or pathogenesis of the disease [30]. Asthma reflects the immune response mounted by Th2 lymphocytes and the failure of regulatory T cells to maintain this response within a normal range. Many varieties of treatment strategies aim to reduce the signs and improve the clinical outcomes of patients. Many research papers have compared the effect of these strategies on asthma patients of different genetic predispositions, ages, genders, environments, and many other factors that can affect the immune response of individual patients. The most important conclusion of all of these studies supports the use of immunotherapy for long-term highly effective treatment. Thus, a better understanding of the immune mechanism through which our bodies can respond to HDM-allergic asthma has great relevance in establishing more efficient and long-term treatment. At the point of enrollment, patients with similar disease circumstances were chosen to exclude confounding factors regarding environmental exposures, the control of asthma and its severity, living conditions, psychosocial circumstances, housing, etc. Moreover, we excluded patients who were taking systemic medications to control their symptoms, which could affect our study results. We did not include severe asthma patients who may need such treatment. Subcutaneous immunotherapy is a proven safe allergic asthma therapy that reduces exacerbations and the need for medications. The Global Initiative for Asthma (GINA) guidelines acknowledge and recommend allergenic immunotherapy (AIT) for asthma treatment. It was clear that symptoms, bronchial hyperreactivity, and drug scores were significantly reduced after 4 months of desensitization using an extract of the house dust mite, *D. pteronyssinus* [31,32]. Our results were compatible with some previous findings and showed improvement in pulmonary functions in the group who received SCIT. The emergence of anti-IgE medications has shown the significance of this type of immunoglobulin in the pathogenesis of allergic disorders. This type of treatment is efficient in the treatment of many allergic disorders such as allergic asthma and rhinitis [33,34]. Immunoglobulin E is a unique class of immunoglobulins that has the lowest blood serum concentration [35]. The local or systemic distribution of IgE grants a sensory mechanism that makes it more sensitive to histamine-secreting mast cells and basophils. Thus, a fast and specific memory response against revisiting antigens takes place that leads to serious allergic disorder [36]. The findings of this study revealed that exposure to HDM aeroallergens in the HDM/AA group dramatically boosted the total serum IgE level to a higher level, which was slightly reduced in the HDM/SCIT group who were receiving immunotherapy. It was reported that up to 25–30% of the world’s population has IgE sensitization to HDM, which is considered as a main risk factor for HDM-allergic asthma [2]. HDM-allergic asthma is one of three high-IgE asthma disorders where there is an inherited tendency to produce high serum IgE levels which suggests that IgE plays an important role in the pathogenesis of asthma [37]. The dose of HDM aeroallergens influences the total serum level of IgE. As previously stated, high doses of house dust mite aeroallergens strikingly elevate total serum IgE levels [38].

The findings of the current study showed significant overexpression of the FoxO1 gene in HDM/SCIT patients who were treated with immunotherapy. Compatible results of another study reported that gene expression of FoxO1 was overexpressed in pulmonary macrophages in patients who were exposed to house dust mite allergens and had mild asthma [39]. In our case, the overexpression of FoxO1 can be correlated to the positive effect of subcutaneous immunotherapy. Immunotherapy treatment stimulates FoxO1 gene expression which improves the proliferation of regulatory T cells to control allergic responses and consequently improve clinical symptoms [40,41,42]. FoxO1 deletion results in the failure of FoxP3 to stimulate regulatory T-cell differentiation [43,44]. Regulatory T cells are a class of T lymphocytes that are responsible for maintaining the immune response within a normal range and are considered as negative regulators of the immune system. Regulatory T cells that are deficient in FoxO1 are pro-inflammatory and develop autoimmune diseases [42]. Downstream regulators of FoxO1 represent a novel control strategy for allergic asthma and the remodeling of airway inflammation [42,45]. SIRT1 gene expression was negatively affected after exposure to HDM aeroallergens in the HDM/AA group. This result is compatible with what is reported by other research studies as the SIRT1 level decreased in peripheral blood mononuclear cells (PBMCs) and lung macrophages in asthmatic lung tissue [46,47]. Some studies found a decrease in serum SIRT1 levels of patients with severe asthma and others found no difference in serum levels between patients with mild, moderate, or severe asthma [48,49]. In contrast, other studies recorded an increase in serum SIRT1 levels that affected pulmonary function [50,51]. Tsilogianni et al. found no relation between pulmonary function and serum SIRT1 levels. However, SIRT1 expression was strikingly elevated in the HDM/SCIT group of the present study, the members of which were subjected to immunotherapy treatment. SIRT1 modulation by activation is an attractive asthma therapeutic strategy. SIRT1 is considered an auxiliary index for diagnosis and its activators represent novel therapeutic strategies for asthma [52]. Moreover, SIRT1 upregulators have protective anti-inflammatory and alleviating roles in asthma [53,54,55]. On the other hand, other findings claimed that SIRT1 plays a pro-inflammation role and promotes airway inflammation in asthma [56,57]. Thus, its inhibition has an effective alleviating impact on pulmonary inflammation and the progression of asthma [58,59,60]. The disparity in the results is due to the controversial role of SIRT1 in asthma. 

Studying the correlation between different immunity biomarkers helps to understand their interplay and their feasible role in the remission of HDM-allergic asthma. There was a negative correlation between total serum IgE levels and FoxO1 expression in HDM-allergic asthma patients whether or not they had received immunotherapy treatment. There was a negative correlation with SIRT1 in HDM-allergic asthma patients who had not received immunotherapy treatment. The same findings were recorded by Colley et al. who stated that the inhibition of SIRT1 function was negatively correlated with total serum IgE [47]. Controversially, other studies concluded that there is a positive correlation between both biomarkers in allergic asthma patients [52]. On the other hand, total serum IgE is positively correlated with SIRT1 in the HDM/SCIT group, the members of which received immunotherapy treatment. In conclusion, the alleviating effect of subcutaneous immunotherapy is declared by its remission effect on clinical symptoms, total IgE levels, and FoxO1 and SIRT1 gene expression in patients who received immunotherapy before recruitment in this study. More rigorous broad-scale studies are needed to determine the role of SIRT1 in the pathogenesis of asthma. Moreover, the results recommend further studies on the use of FoxO1 and SIRT1 in the treatment of HDM-allergic asthma.

## 5. Limitations

The interactions between the investigated immune markers and their role in either provoking or controlling inappropriate immune reactions are still foggy. Different research papers have opposing results due to many factors such as genetic ancestry, age, gender, severity of asthma, and type of immunotherapy. In addition, the low number of recruited patients in every study is one of the limitations that hinder trusted results.

## 6. Conclusions

HDM-allergic asthma is one of the most common allergic disorders that affects human performance and quality of life, especially at younger ages. Studying different immune biomarkers and comparing their expression between healthy subjects and allergic asthma patients who did or did not receive immunotherapy, the best known treatment at the moment, give valuable knowledge for promising plans for asthma therapy and the remission of symptoms. Broader-scale research studies are needed to elucidate the immune mechanisms in response to allergic disorders to discover novel immune-based therapeutic strategies.

## Figures and Tables

**Table 1:** Primer sequences for the FoxO1, SIRT1, and 18S rRNA genes.

Primer	Sense Primer Sequence	Antisense Primer	Product Size (bp)
FoxO1	5′-GTCAAGAGCGTGCCCTACTTCA-3′	5′-TGAACTTGCTGTGTAGGGACAGATTAT-3′	102 [22]
SIRT1	5′-TGCTGGCCTAATAGAGTGGCA-3′	5′-CTCAGCGCCATGGAAAATGT-3′	101 [23]
18S rRNA	5′-AGT CCC TGC CCT TTG TAC ACA-3′	5′-GAT CCG AGG GCC TCA CTA AAC-3′	69 [24]

**Table 2:** PCR thermocycler conditions of RT-PCR of FoxO1, SIRT1, and 18s rRNA.

PCR Step	Temperature	Time	Repeat Cycles
Initial denaturation	95 °C	10 min	
Denaturation	95 °C	15 s	40
Annealing	60 °C	30 s	
Extension	72 °C	30 s	30

**Table 3:** Criteria of study participants.

Criteria	HV *n* = 40	HDM/AA *n* = 40	HDM/SCIT *n* = 40	F	*p* Value
AGE Mean ± SD	30.75 ± 9.8	29.60 ± 11.5	34.08 ± 12.3	0.337	ns ^$^
Gender male/female	17/23 (42.5/57.5%)	18/22 (45/55%)	20/20 (50/50%)	-	
Residence Urban Rural	25 (62%) 15 (37.5%)	22 (55%) 18 (45%)	23 (57.5%) 17 (42.5%)	-	
Family history of asthma Negative Positive	31 (77.5%) 9 (22.5%)	8 (20%) 32 (80%)	9 (22.5%) 31 (77.5%)	-	
Skin prick test	0%	100%	100%	-	
House dust mites *D. pteronyssinus* *D. farinae* Both	0% - - -	100% 28 24 24	100% 27 25 25	-	
Severity of asthma Grade I Grade II		10 30	29 11	-	
FEV1 (% of predicted)	91.10 ± 9.666	72.62 ± 4.943	87.49 ± 2.52	-	<0.0001 **^#^
FEV1/FVC (%)	91.91 ± 1.849	71.14 ± 2.141	88.59 ± 8.247	-	<0.0001 **^#^
Total serum IgE (ng/mL) Mean ± SD	158.3 ± 73.1	921.3 ± 317.3	798.2 ± 204.0	-	<0.0001 **^#^
FoxO1 gene expression (ng/mL) Mean ± SD	1.29 ± 0.76	1.33 ± 0.43	1.82 ± 0.49	-	<0.0001 **^#^
SIRT1 gene expression (ng/mL) Mean ± SD	1.6 ± 0.52	1.2 ± 0.31	2.03 ± 0.43	41.59	<0.0001 **^$^

** significant difference. *p* ≤ 0.05 is statistically significant. ^$^: one-way ANOVA test. ^#^: Kruskal–Wallis test.

**Table 4:** Multiple comparisons between the study groups.

Compared Groups	HV vs. HDM/AA	HV vs. HDM/SCIT	HDM/AA vs. HDM/SCIT
FEV ^#^	<0.0001 **	0.1679 ^ns^	<0.0001 **
FEV1/FVC% ^#^	<0.0001 **	>0.9999 ^ns^	<0.0001 **
Total serum IgE (ng/mL) ^#^	<0.0001 **	<0.0001 **	>0.9999 ^ns^
FoxO1 gene expression (ng/mL) ^#^	>0.9999 ^ns^	<0.0001 **	0.0004 **
Sirtuin 1 gene expression (ng/mL) ^$^	0.0002 **	<0.0001 **	<0.0001 **

** significant difference. ^ns^: non-significant. *p* ≤ 0.05 is statistically highly significant. ^$^: one-way ANOVA test. ^#^: Kruskal–Wallis test.

**Table 5:** Correlation between total serum IgE and FoxO1 and SIRT1 expression in different study groups.

	HV		HDM/AA		HDM/SCIT	
Characteristics	*r*	*p*-Value	*r*	*p*-Value	*r*	*p*-Value
FoxO1 (ng/mL)	0.4338	0.0052 **	−0.3108	0.0510 ^ns^	−0.01030	0.9497 ^ns^
SIRT1 (ng/mL)	−0.1120	0.4915 ^ns^	−0.3321	0.0363 *	0.08908	0.5847 ^ns^

^ns^—non-significant. * *p* ≤ 0.05, ** *p* ≤ 0.005.

## Data Availability

All data generated or analyzed during the current study are included in this article.

## References

[1] Ando T., Kitaura J. (2021). Tuning IgE: IgE-Associating Molecules and Their Effects on IgE-Dependent Mast Cell Reactions. Cells.

[2] Arlian L.G., Bernstein D., Bernstein I.L., Friedman S., Grant A., Lieberman P., Lopez M., Metzger J., Platts-Mills T., Schatz M. (1992). Prevalence of dust mites in the homes of people with asthma living in eight different geographic areas of the United States. J. Allergy Clin. Immunol..

[3] Australasian Society of Clinical Immunology and Allergy (ASCIA) (2020). Skin Prick Testing Guide for Diagnosis of Allergic Disease. https://www.allergy.org.au/images/ASCIA_HP_SPT_Guide_2020.pdf.

[4] Bachert C., Zhang N. (2012). Chronic rhinosinusitis and asthma: Novel understanding of the role of IgE ‘above atopy’. J. Intern. Med..

[5] Baioumy S.A., Elgendy A., Ibrahim S.M., Taha S.I., Fouad S.H. (2021). Association between serum zonulin level and severity of house dust mite allergic asthma. Allergy Asthma Clin. Immunol..

[6] Beeh K.M., Ksoll M., Buhl R. (2000). Elevation of total serum immunoglobulin E is associated with asthma in nonallergic individuals. Eur. Respir. J..

[7] Bernstein I.L., Li J.T., Bernstein D.I., Hamilton R., Spector S.L., Tan R., Sicherer S., Golden D.B.K., Khan D.A., Nicklas R.A. (2008). Allergy Diagnostic Testing: An Updated Practice Parameter. Ann. Allergy Asthma Immunol..

[8] Bijanzadeh M., Mahesh P.A., Ramachandra N.B. (2011). An understanding of the genetic basis of asthma. Indian J. Med. Res..

[9] Blank U., Huang H., Kawakami T. (2021). The high affinity IgE receptor: A signaling update. Curr. Opin. Immunol..

[10] de Blay F., Gherasim A., Casale T.B., Doyen V., Bernstein D. (2022). Which patients with asthma are most likely to benefit from allergen immunotherapy?. J. Allergy Clin. Immunol..

[11] Blink S.E., Fu Y.-X. (2010). IgE regulates T helper cell differentiation through FcγRIII mediated dendritic cell cytokine modulation. Cell. Immunol..

[12] Brunet A., Sweeney L.B., Sturgill J.F., Chua K.F., Greer P.L., Lin Y., Tran H., Ross S.E., Mostoslavsky R., Cohen H.Y. (2004). Stress-Dependent Regulation of FOXO Transcription Factors by the SIRT1 Deacetylase. Science.

[13] Burrows B., Martinez F.D., Halonen M., Barbee R.A., Cline M.G. (1989). Association of Asthma with Serum IgE Levels and Skin-Test Reactivity to Allergens. N. Engl. J. Med..

[14] Chen C.A., Wallace C.C., Wolstenholme J. (2002). Analysis of the mitochondrial 12S rRNA gene supports a two-clade hypothesis of the evolutionary history of scleractinian corals. Mol. Phylogenet. Evol..

[15] Chen X., Sun K., Jiao S., Cai N., Zhao X., Zou H., Xie Y., Wang Z., Zhong M., Wei L. (2014). High levels of SIRT1 expression enhance tumorigenesis and associate with a poor prognosis of colorectal carcinoma patients. Sci. Rep..

[16] Childs C.E., Munblit D., Ulfman L., Gómez-Gallego C., Lehtoranta L., Recker T., Salminen S., Tiemessen M., Collado M.C. (2022). Potential Biomarkers, Risk Factors, and Their Associations with IgE-Mediated Food Allergy in Early Life: A Narrative Review. Adv. Nutr..

[17] Gandhi P.K., Kenzik K.M., Thompson L.A., DeWalt D.A., Revicki D.A., Shenkman E.A., Huang I.-C. (2013). Exploring factors influencing asthma control and asthma-specific health-related quality of life among children. Respir. Res..

[18] Chung S., Kim J.Y., Song M.A., Park G.Y., Lee Y.G., Karpurapu M., Englert J.A., Ballinger M.N., Pabla N., Chung H.Y. (2019). FoxO1 is a critical regulator of M2-like macrophage activation in allergic asthma. Allergy Eur. J. Allergy Clin. Immunol..

[19] Colley T., Mercado N., Kunori Y., Brightling C., Bhavsar P.K., Barnes P.J., Ito K. (2016). Defective sirtuin-1 increases IL-4 expression through acetylation of GATA-3 in patients with severe asthma. J. Allergy Clin. Immunol..

[20] Custovic A. (2015). To what extent is allergen exposure a risk factor for the development of allergic disease?. Clin. Exp. Allergy.

[21] Dilmac S., Kuscu N., Caner A., Yildirim S., Yoldas B., Farooqi A.A., Tanriover G. (2022). SIRT1/FOXO Signaling Pathway in Breast Cancer Progression and Metastasis. Int. J. Mol. Sci..

[22] Du X., Shi H., Li J., Dong Y., Liang J., Ye J., Kong S., Zhang S., Zhong T., Yuan Z. (2014). Mst1/Mst2 Regulate Development and Function of Regulatory T Cells through Modulation of Foxo1/Foxo3 Stability in Autoimmune Disease. J. Immunol..

[23] Farhan M., Wang H., Gaur U., Little P.J., Xu J., Zheng W. (2017). FOXO Signaling Pathways as Therapeutic Targets in Cancer. Int. J. Biol. Sci..

[24] Froidure A., Mouthuy J., Durham S.R., Chanez P., Sibille Y., Pilette C. (2016). Asthma phenotypes and IgE responses. Eur. Respir. J..

[25] National Institutes of Health (2020). Global Initiative for Asthma—GINA. Global Strategy for Asthma Management and Prevention Revision. https://ginasthma.org/.

[26] Graves D.T., Milovanova T.N. (2019). Mucosal Immunity and the FOXO1 Transcription Factors. Front. Immunol..

[27] Guan R., Cai Z., Wang J., Ding M., Li Z., Xu J., Li Y., Li J., Yao H., Liu W. (2019). Hydrogen sulfide attenuates mitochondrial dysfunction-induced cellular senescence and apoptosis in alveolar epithelial cells by upregulating sirtuin 1. Aging.

[28] Harada Y., Harada Y., Elly C., Ying G., Paik J.-H., DePinho R.A., Liu Y.-C. (2010). Transcription factors Foxo3a and Foxo1 couple the E3 ligase Cbl-b to the induction of Foxp3 expression in induced regulatory T cells. J. Exp. Med..

[29] Hsu P., Santner-Nanan B., Hu M., Skarratt K., Lee C.H., Stormon M., Wong M., Fuller S.J., Nanan R. (2015). IL-10 Potentiates Differentiation of Human Induced Regulatory T Cells via STAT3 and Foxo1. J. Immunol..

[30] Humbert M., Durham S.R., Ying S., Kimmitt P., Barkans J., Assoufi B., Pfister R., Menz G., Robinson D.S., Kay A.B. (1996). IL-4 and IL-5 mRNA and protein in bronchial biopsies from patients with atopic and nonatopic asthma: Evidence against “intrinsic” asthma being a distinct immunopathologic entity. Am. J. Respir. Crit. Care Med..

[31] Ichikawa T., Hayashi R., Suzuki K., Imanishi S., Kambara K., Okazawa S., Inomata M., Yamada T., Yamazaki Y., Koshimizu Y. (2013). Sirtuin 1 activator SRT1720 suppresses inflammation in an ovalbumin-induced mouse model of asthma. Respirology.

[32] Jiang Y., Deng S., Hu X., Luo L., Zhang Y., Zhang D., Li X., Feng J. (2022). Identification of potential biomarkers and immune infiltration characteristics in severe asthma. Int. J. Immunopathol. Pharmacol..

[33] Kawakami T., Galli S.J. (2002). Regulation of mast-cell and basophil function and survival by IgE. Nat. Rev. Immunol..

[34] Khan M.A. (2020). Regulatory T cells mediated immunomodulation during asthma: A therapeutic standpoint. J. Transl. Med..

[35] Kim S.R., Lee K.S., Park S.J., Min K.H., Choe Y.H., Moon H., Yoo W.H., Chae H.-J., Han M.K., Lee Y.C. (2010). Involvement of sirtuin 1 in airway inflammation and hyperresponsiveness of allergic airway disease. J. Allergy Clin. Immunol..

[36] Lallemand F., Vacher S., de Koning L., Petitalot A., Briaux A., Driouch K., Callens C., Schnitzler A., Lecerf C., Oulie-Bard F. (2020). The high protein expression of FOXO3, but not that of FOXO1, is associated with markers of good prognosis. Sci. Rep..

[37] Lee J., Kim J., Lee J.H., Choi Y.M., Choi H., Cho H.D., Cha G.H., Lee Y.H., Jo E.K., Park B.H. (2022). SIRT1 Promotes Host Protective Immunity against Toxoplasma gondii by Controlling the FoxO-Autophagy Axis via the AMPK and PI3K/AKT Signalling Pathways. Int. J. Mol. Sci..

[38] Luo G., Jian Z., Zhu Y., Zhu Y., Chen B., Ma R., Tang F., Xiao Y. (2019). Sirt1 promotes autophagy and inhibits apoptosis to protect cardiomyocytes from hypoxic stress. Int. J. Mol. Med..

[39] McKnight C.G., Jude J.A., Zhu Z., Panettieri R.A., Finkelman F.D. (2017). House dust mite-induced allergic airway disease is independent of IgE and FceRIa. Am. J. Respir. Cell Mol. Biol..

[40] Medema R.H., Jäättelä M. (2010). Cytosolic FoxO1: Alive and killing. Nat. Cell Biol..

[41] Ng F., Tang B.L. (2013). Sirtuins’ modulation of autophagy. J. Cell. Physiol..

[42] Oettgen H.C., Geha R.S. (1999). IgE in asthma and atopy: Cellular and molecular connections. J. Clin. Investig..

[43] Ouyang W., Beckett O., Flavell R.A., Li M.O. (2009). An Essential Role of the Forkhead-Box Transcription Factor Foxo1 in Control of T Cell Homeostasis and Tolerance. Immunity.

[44] Ouyang W., Beckett O., Ma Q., Paik J., DePinho R.A., Li M.O. (2010). Foxo proteins cooperatively control the differentiation of Foxp3+ regulatory T cells. Nat. Immunol..

[45] Ouyang W., Liao W., Luo C.T., Yin N., Huse M., Kim M.V., Peng M., Chan P., Ma Q., Mo Y. (2012). Novel Foxo1-dependent transcriptional programs control Treg cell function. Nature.

[46] Qiu C., Zhong L., Huang C., Long J., Ye X., Wu J., Dai W., Lv W., Xie C., Zhang J. (2020). Cell-bound IgE and plasma IgE as a combined clinical diagnostic indicator for allergic patients. Sci. Rep..

[47] Starkl P., Marichal T., Gaudenzio N., Reber L.L., Sibilano R., Tsai M., Galli S.J. (2016). IgE antibodies, FcεRIα, and IgE-mediated local anaphylaxis can limit snake venom toxicity. J. Allergy Clin. Immunol..

[48] Tang L., Chen Q., Meng Z., Sun L., Zhu L., Liu J., Hu J., Ni Z., Wang X. (2017). Suppression of Sirtuin-1 Increases IL-6 Expression by Activation of the Akt Pathway During Allergic Asthma. Cell. Physiol. Biochem..

[49] Tsabouri S., Mavroudi A., Feketea G., Guibas G.V. (2017). Subcutaneous and Sublingual Immunotherapy in Allergic Asthma in Children. Front. Pediatr..

[50] Tsilogianni Z., Baker J.R., Papaporfyriou A., Papaioannou A.I., Papathanasiou E., Koulouris N.G., Daly L., Ito K., Hillas G., Papiris S. (2020). Sirtuin 1: Endocan and Sestrin 2 in Different Biological Samples in Patients with Asthma. Does Severity Make the Difference?. J. Clin. Med..

[51] Vandenplas O. (2011). Reduction of exposure in the management of occupational asthma. Curr. Opin. Allergy Clin. Immunol..

[52] Wang Y., Li D., Ma G., Li W., Wu J., Lai T., Huang D., Zhao X., Lv Q., Chen M. (2015). Increases in peripheral SIRT1: A new biological characteristic of asthma. Respirology.

[53] Wu Y., Li W., Hu Y., Liu Y., Sun X. (2020). Suppression of sirtuin 1 alleviates airway inflammation through mTOR-mediated autophagy. Mol. Med. Rep..

[54] Yu J., Liu X., Li Y., Meng S., Wu F., Yan B., Xue Y., Ma T., Yang J., Liu J. (2018). Maternal exposure to farming environment protects offspring against allergic diseases by modulating the neonatal TLR-Tregs-Th axis. Clin. Transl. Allergy.

[55] Zhang H., Sun Y., Rong W., Fan L., Cai Y., Qu Q., Gao Y., Zhao H. (2018). miR-221 participates in the airway epithelial cells injury in asthma via targeting SIRT1. Exp. Lung Res..

[56] Zhang X.-Y., Li W., Zhang J.-R., Li C.-Y., Zhang J., Lv X.-J. (2022). Roles of sirtuin family members in chronic obstructive pulmonary disease. Respir. Res..

[57] Zhou Y., Zhang F., Ding J. (2022). As a Modulator, Multitasking Roles of SIRT1 in Respiratory Diseases. Immune Netw..

[58] Ziyaei T., Berenji F., Jabbari-Azad F., Fata A., Jarahi L., Fereidouni M. (2017). House Dust Mite Prevalence in the House of Patients with Atopic Dermatitis in Mashhad. Iran. J Arthropod-Borne Dis..

[59] Zou B., Fu Y., Cao C., Pan D., Wang W., Kong L. (2021). Gentiopicroside ameliorates ovalbumin-induced airway inflammation in a mouse model of allergic asthma via regulating SIRT1/NF-κB signaling pathway. Pulm. Pharmacol. Ther..

[60] Biomarkers Definitions Working Group (2001). Biomarkers and surrogate endpoints: Preferred definitions and conceptual framework. Clin. Pharmacol. Ther..

